# Disaggregation Following Agonist-Induced Platelet Activation in Patients on Dual Antiplatelet Therapy

**DOI:** 10.1007/s12265-017-9746-0

**Published:** 2017-04-19

**Authors:** Patricia P. Wadowski, Beate Eichelberger, Christoph W. Kopp, Joseph Pultar, Daniela Seidinger, Renate Koppensteiner, Irene M. Lang, Simon Panzer, Thomas Gremmel

**Affiliations:** 1 Division of Angiology, Department of Internal Medicine II, Medical University of Vienna, Waehringer Guertel 18-20, 1090 Vienna, Austria; 2 Department of Blood Group Serology and Transfusion Medicine, Medical University of Vienna, Vienna, Austria; 3 Division of Cardiology, Department of Internal Medicine II, Medical University of Vienna, Vienna, Austria

**Keywords:** Light transmission aggregometry, Platelet disaggregation, High residual platelet reactivity, Clopidogrel, Prasugrel, Ticagrelor

## Abstract

Disaggregation as the difference between maximal and final platelet aggregation by light transmission aggregometry indicates the stability of platelet aggregates. We evaluated the extent of disaggregation after platelet stimulation with adenosine diphosphate (ADP), arachidonic acid (AA), collagen, epinephrine, and thrombin receptor-activating peptide (TRAP)-6 in 323 patients on dual antiplatelet therapy with daily aspirin and clopidogrel (group 1), prasugrel (group 2), or ticagrelor (group 3) therapy. All patients in group 1 underwent elective angioplasty and stenting, whereas all patients included in groups 2 and 3 suffered from acute coronary syndromes (STEMI or NSTEMI) and underwent urgent PCI. Significant differences between maximal and final platelet aggregation were observed with all agonists throughout the groups (all *p*<0.001). Disaggregation was highest using AA (clopidogrel 36.5%; prasugrel/ticagrelor 100%) and ADP (clopidogrel 21.7%; prasugrel/ticagrelor 100%). In contrast, low disaggregation was observed after platelet stimulation with collagen and TRAP-6 in clopidogrel-treated patients, and after platelet stimulation with collagen and epinephrine in prasugrel- and ticagrelor-treated patients. In conclusion, pathways of platelet activation that are not inhibited by standard antiplatelet therapy allow persisting platelet aggregation and may at least in part be responsible for adverse ischemic events.

## Introduction

Platelet function tests are increasingly performed to monitor patients’ response to antiplatelet therapy. Thereby, different measurement principles can be applied, i.e., tests assessing surrogate markers of platelet aggregation (light transmission aggregometry, VerifyNow assay, impedance aggregometry, Plateletworks) or platelet adhesion under high shear stress (platelet function analyzer-100, Cone and Plate(let) Analyzer), viscoelastic measurements (thromboelastography, platelet mapping and ROTEM), flow cytometry, and evaluation of thromboxane metabolites (radio- or enzyme-linked immunoassays). [1–3]

Light transmission aggregometry (LTA) was described independently by Born [4] and O’Brien [5] in the 1960s for the in vitro assessment of platelet function. A variety of agonists can be used for this purpose, with the most popular being adenosine diphosphate (ADP), collagen, arachidonic acid (AA), epinephrine, and thrombin receptor-activating peptide (TRAP)-6. [6] Platelet aggregation following the in vitro activation by agonists became a widely used method to assess the response to antiplatelet therapy. [7] High on-treatment residual platelet reactivity (HRPR) to ADP in patients with the P2Y_12_ receptor inhibitor clopidogrel as determined by LTA has repeatedly been associated with adverse clinical outcomes after percutaneous coronary intervention (PCI) with stent implantation. [8, 9] Normal aggregation curves can be seen using platelet-rich plasma (PRP) from subjects without antiplatelet therapy but the extent of persisting stable aggregates after maximal aggregation varies. [4, 6] This phenomenon is augmented in patients on clopidogrel therapy. [10] In detail, the difference between maximal and final aggregation by LTA indicates the stability of platelet aggregates. A small difference means low disaggregation, whereas a great difference between the two values reflects high disaggregation, i.e., a low stability of platelet aggregates.

So far, there are no data on the extent of disaggregation after addition of different agonists to platelets from individuals on dual antiplatelet therapy (DAPT) with aspirin and an ADP P2Y_12_ receptor blocker. In order to study the stability of platelet aggregate formation in response to various agonists, we investigated the differences in maximal and final platelet aggregation by LTA using ADP, collagen, epinephrine, and TRAP-6 in samples from patients on DAPT with aspirin and clopidogrel, prasugrel, or ticagrelor. These data should provide a better understanding of active pathways in platelets inhibited by current DAPT.

## Methods

### Patients

Light transmission aggregometry was performed in the following three groups: group 1, 256 patients on daily aspirin (100 mg/day) and clopidogrel (75 mg/day) therapy were included following elective percutaneous intervention with endovascular stent implantation. Groups 2 and 3, 67 patients on daily aspirin (100 mg/day) and prasugrel (10 mg/d, *n* = 47) or ticagrelor (180 mg/day, *n* = 20) therapy were included following acute coronary syndrome with PCI and endovascular stent implantation. Blood sampling was performed 24 h after elective and 72 h after acute percutaneous interventions.

In group 1, 69 patients (27%) were on chronic clopidogrel therapy (75 mg/day) and therefore not loaded, 115 patients (44.9%) received a loading dose of 300 mg clopidogrel 24 h before the intervention, and 72 patients (28.1%) received a loading dose of 600 mg clopidogrel on the day of the percutaneous procedure at least 2 h before the intervention.

In group 2, all patients were loaded with 60 mg prasugrel followed by 10 mg prasugrel/day and in group 3 all patients were loaded with 180 mg ticagrelor followed by 90 mg ticagrelor twice daily.

All patients in group 1 underwent elective angioplasty and stenting, whereas all patients included in groups 2 and 3 suffered from acute coronary syndromes (STEMI or NSTEMI) and underwent urgent PCI.

Exclusion criteria were a known aspirin, clopidogrel, prasugrel, or ticagrelor intolerance (allergic reactions, gastrointestinal bleeding); an ongoing treatment with vitamin K antagonists (warfarin, phenprocoumon, acenocoumarol) or direct oral anticoagulants (dabigatran, rivaroxaban, apixaban, edoxaban); a treatment with glycoprotein IIb/IIIa inhibitors; a treatment with ticlopidine, dipyridamol, or non-steroidal anti-inflammatory drugs; a family or personal history of bleeding disorders, malignant paraproteinemias, myeloproliferative disorders, or heparin-induced thrombocytopenia; severe hepatic failure; known qualitative defects in thrombocyte function; a major surgical procedure within 1 week before enrollment; a platelet count <100.000 or >450.000/μl; and a hematocrit <30%.

The study was performed in accordance with the Declaration of Helsinki and approved by the local Ethics Committee of the Medical University of Vienna. All study participants signed a written informed consent.

### Light Transmission Aggregometry

Light transmission aggregometry (LTA) was performed on a PAP-8E aggregometer (Bio-Data, Horsham, PA USA) as previously described. [2] Citrate-anticoagulated whole blood was allowed to “rest” in a tilt position at room temperature for 20 min before centrifugation. Blood tubes were centrifuged at 150×*g* for 10 min (min) at room temperature to acquire PRP. To obtain platelet-poor plasma (PPP), remaining specimens were re-centrifugated at 2000×*g* for 10 min. Platelet counts were not adjusted as the median platelet count was 210 G/l (range 177–250 G/l) for group 1, 195 G/l (range 164–239 G/l) for group 2, and 190 G/l (range 144–240 G/l) for group 3. The baseline optical density was set with PPP. Platelet aggregation was initiated by the following agonists: ADP (10 μM), AA (0.5 mg/dl), epinephrine (5.5 μM), collagen (190 μg/ml), and TRAP-6 (25 μM). Optical density changes were recorded photoelectrically for 10 min as platelets began to aggregate to obtain maximal and final aggregation values.

### Statistics

Statistical analyses were performed using IBM SPSS Statistics for Macintosh, Version 21.0. (IBM Corp. Armonk, NY, Released 2012). Median and interquartile range of continuous variables are shown. Aggregation data were described as median and interquartile range and differences analyzed with the non-parametric Wilcoxon signed-rank test. Two-sided *p* values <0.05 were considered statistically significant.

Disaggregation in percent was calculated using the following formula: [(maximal aggregation − final aggregation)/maximal aggregation] × 100. Boxplots were used to depict disaggregation values in percent between the four agonists.

## Results

Clinical, laboratory, and procedural characteristics of the patients are given in Table 1. As expected, clopidogrel- and ticagrelor-treated patients were significantly older than prasugrel-treated patients. Moreover, hypertension and previous myocardial infarction were more common in clopidogrel-treated patients whereas male sex and hyperlipidemia were more common in patients treated with ticagrelor or prasugrel. Finally, we found significant intergroup differences regarding the use of beta blockers, calcium channel blockers, angiotensin-converting enzyme inhibitors, and angiotensin receptor blockers.

**Table 1:** Clinical, laboratory, and procedural characteristics of the patient population

Characteristics	Clopidogrel *n* = 256	Prasugrel *n* = 47	Ticagrelor *n* = 20	*p* value
Age (years)	65 (57–75)	58 (46–64)	65 (56–71)	<0.001
Male sex	170 (66.4%)	40 (85%)	15 (75%)	0.03
BMI (kg/m^2^)	26.6 (24.1–29.4)	27.4 (25.2–29.8)	27.4 (26.3–31.6)	n.s.
Medical history				
Previous myocardial infarction	107 (41.8%)	9 (19.1%)	6 (30.0%)	0.01
Previous TIA/stroke	26 (10.2%)	3 (6.4%)	0 (0%)	n.s.
Hypertension	226 (88.3%)	33 (70.2%)	15 (75.0%)	0.003
Hyperlipidemia	235 (91.8%)	46 (97.9%)	20 (100%)	0.02
Diabetes mellitus	79 (30.9%)	12 (25.5%)	9 (45.0%)	n.s.
-Type I	24 (9.4%)	0 (0%)	1 (5.0%)	n.s.
-Type II	55 (21.5%)	12 (25.5%)	8 (40.0%)	n.s.
Smoking	110 (43.0%)	26 (55.3%)	8 (40.0%)	n.s.
Platelet count (G/l)	210 (177–250)	195 (164–239)	190 (144–240)	n.s.
Stent implantation	256 (100%)	47 (100%)	20 (100%)	n.s.
No. of stents/patient	1 (1–2)	1 (1–2)	1 (1–2)	n.s.
Medication				
Statins	243 (94.9%)	47 (100%)	20 (100%)	n.s.
Beta blockers	173 (67.6%)	47 (100%)	19 (95%)	<0.001
Calcium channel blockers	77 (30.1%)	1 (2.1%)	3 (15.0%)	<0.001
ACE inhibitors	151 (59.0%)	40 (85.1%)	11 (55.0%)	<0.01
Angiotensin receptor blockers	72 (28.1%)	6 (12.8%)	8 (40.0%)	<0.05

Continuous data are presented as median (interquartile range). Dichotomous data are presented as *n* (%)

*BMI* body mass index, *TIA* transient ischemic attack, *ACE* angiotensin-converting enzyme

### Group 1 (Aspirin and Clopidogrel)

**Fig. 1:**
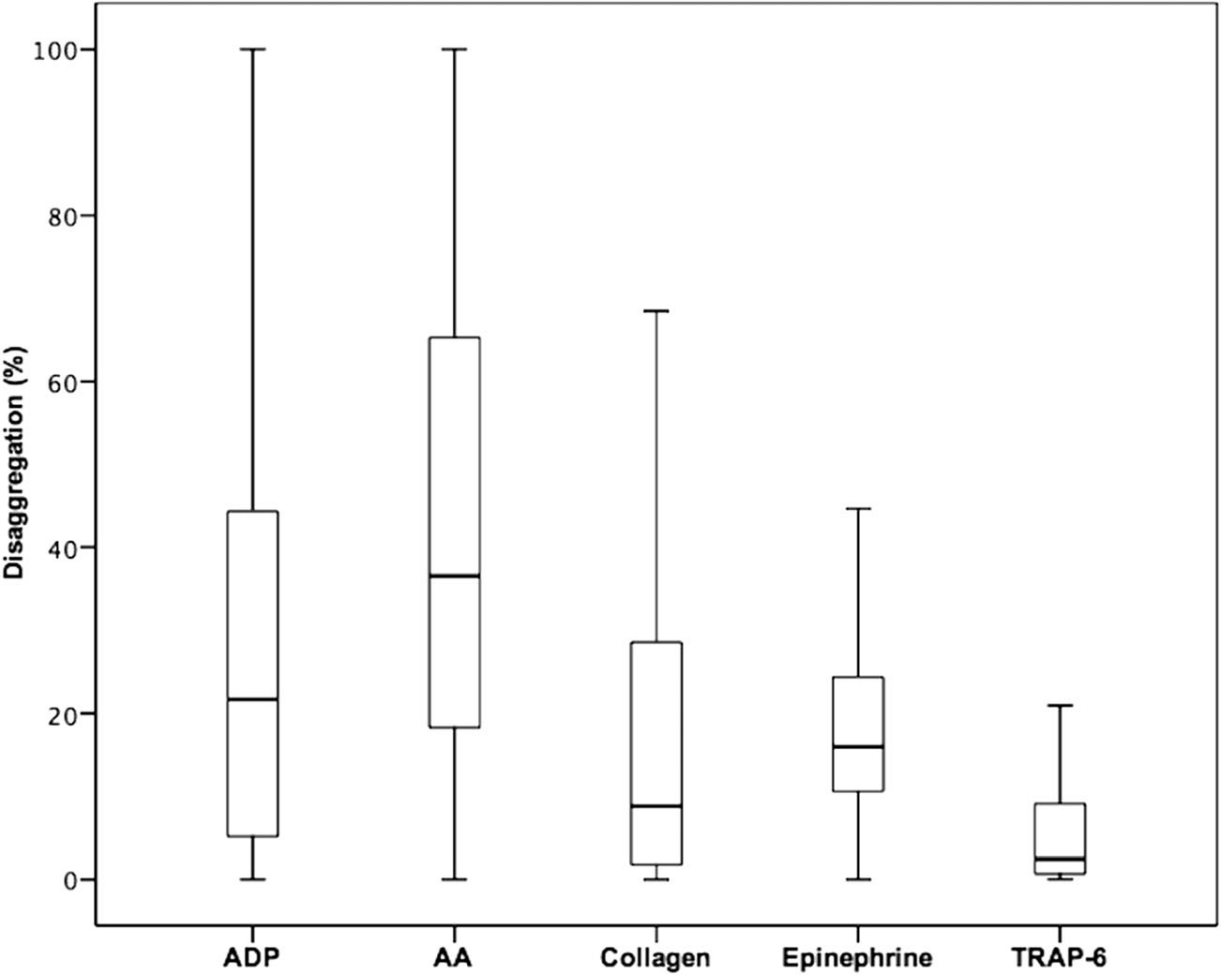
Disaggregation values obtained after stimulation with the agonists arachidonic acid (AA), adenosine diphosphate (ADP), collagen epinephrine, and thrombin receptor-activating peptide-6 (TRAP-6) in patients on aspirin and clopidogrel therapy (*n* = 256). The *boundaries of the box* show the lower and upper quartile of data, and the *line inside the box* represents the median. *Whiskers* are drawn from the edge of the box to the highest and lowest values that are outside the box but within 1.5 times the box length

Significant differences were observed between maximal and final aggregation values using all agonists (Table 2). The extent of disaggregation varied significantly between all agonists (all *p* < 0.05), except between epinephrine and collagen (*p* = 0.2). Median (range) values of disaggregation were highest for arachidonic acid (36.5% (18.2–66.3%); Fig. 1). For ADP, a disaggregation of 21.7% (5.1–44.6%) was obtained and for the agonist epinephrine a difference of 16.0% (10.6–24.4%) was seen. The lowest disaggregation rate was observed after platelet stimulation with collagen (8.8% (1.8–28.9%) and TRAP-6 (2.5% (0.7–9.2%); Fig. 1). As expected, disaggregation following the stimulation with all agonists correlated inversely with the respective final aggregation values (ADP 10 μM: *r* = −0.8; AA: *r* = −0.7; epinephrine: *r* = −0.4; collagen: *r* = −0.7; TRAP-6: *r* = −0.8; all *p* < 0.001).

**Table 2:** Maximal and final platelet aggregation in response to adenosine diphosphate (ADP), arachidonic acid (AA), collagen, epinephrine, and thrombin receptor-activating peptide (TRAP)-6 in clopidogrel-treated patients (*n* = 256)

	Maximal aggregation (%)	Final aggregation (%)	*p* value
ADP	45.6 (33.2–61.3)	31 (17.2–54.4)	<0.001
AA	3.5 (1.4–7.7)	2.3 (0.4–5.5)	<0.001
Collagen	22.2 (9.2–44.0)	18.6 (6.0–40.6)	<0.001
Epinephrine	29.3 (16.2–45)	24.9 (12.6–37.5)	<0.001
TRAP-6	63.2 (48.7–74)	61.4 (43.1–73)	<0.001

Data are presented as median and interquartile range

In a second step, a maximal aggregation >67% in response to ADP was defined as HRPR according to the recent consensus document by Tantry et al. [9] With use of this cut-off value, HRPR ADP was seen in 39 patients (15.2%). Patients with HRPR ADP showed significantly weaker disaggregation after stimulation with ADP, AA, collagen, and TRAP-6 (all *p* ≤ 0.003). Finally, patients with disaggregation in the highest and lowest quartiles following activation with ADP were defined as patients with high and low disaggregation, respectively. Among patients with HRPR ADP, only 1 out of 39 patients (2.6%) showed high disaggregation, whereas 27 patients (69.2%) showed low disaggregation.

Disaggregation following the activation with all agonists correlated inversely with the respective final aggregation values (ADP: *r* = −0.8 (*p* < 0.001); TRAP-6: *r* = −0.8, (*p* < 0.001); AA: *r* = −0.7 (*p* < 0.001); collagen: *r* = −0.7 (*p* < 0.001); epinephrine: *r* = −0.4 (*p* < 0.001)).

### Group 2 (Aspirin and Prasugrel) and Group 3 (Aspirin and Ticagrelor)

**Fig. 2:**
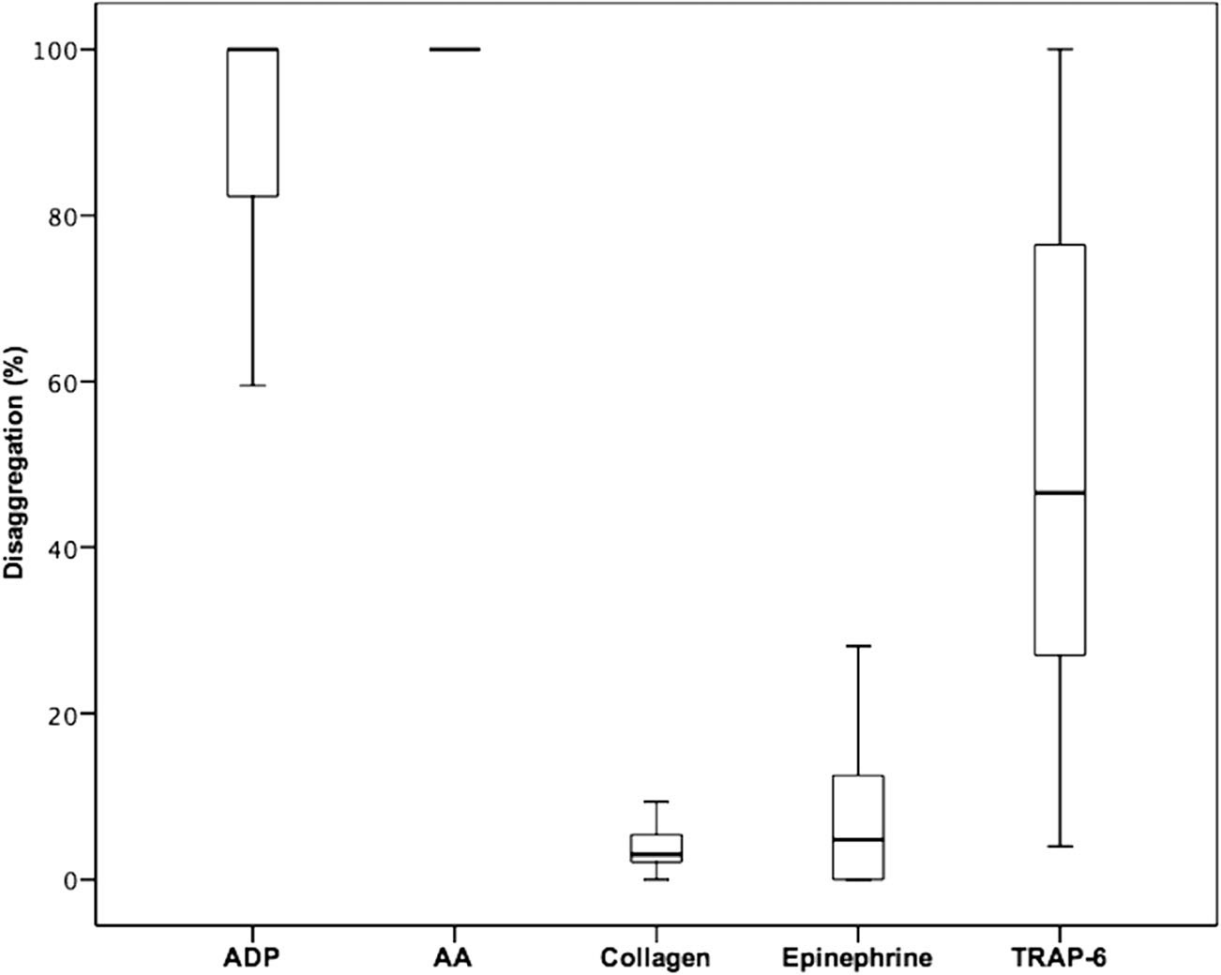
Disaggregation values obtained after stimulation with the agonists arachidonic acid (AA), adenosine diphosphate (ADP), collagen epinephrine, and thrombin receptor-activating peptide-6 (TRAP-6) in patients on aspirin and prasugrel therapy (*n* = 47). The *boundaries of the box* show the lower and upper quartile of data, and the *line inside the box* represents the median. *Whiskers* are drawn from the edge of the box to the highest and lowest values that are outside the box but within 1.5 times the box length

**Fig. 3:**
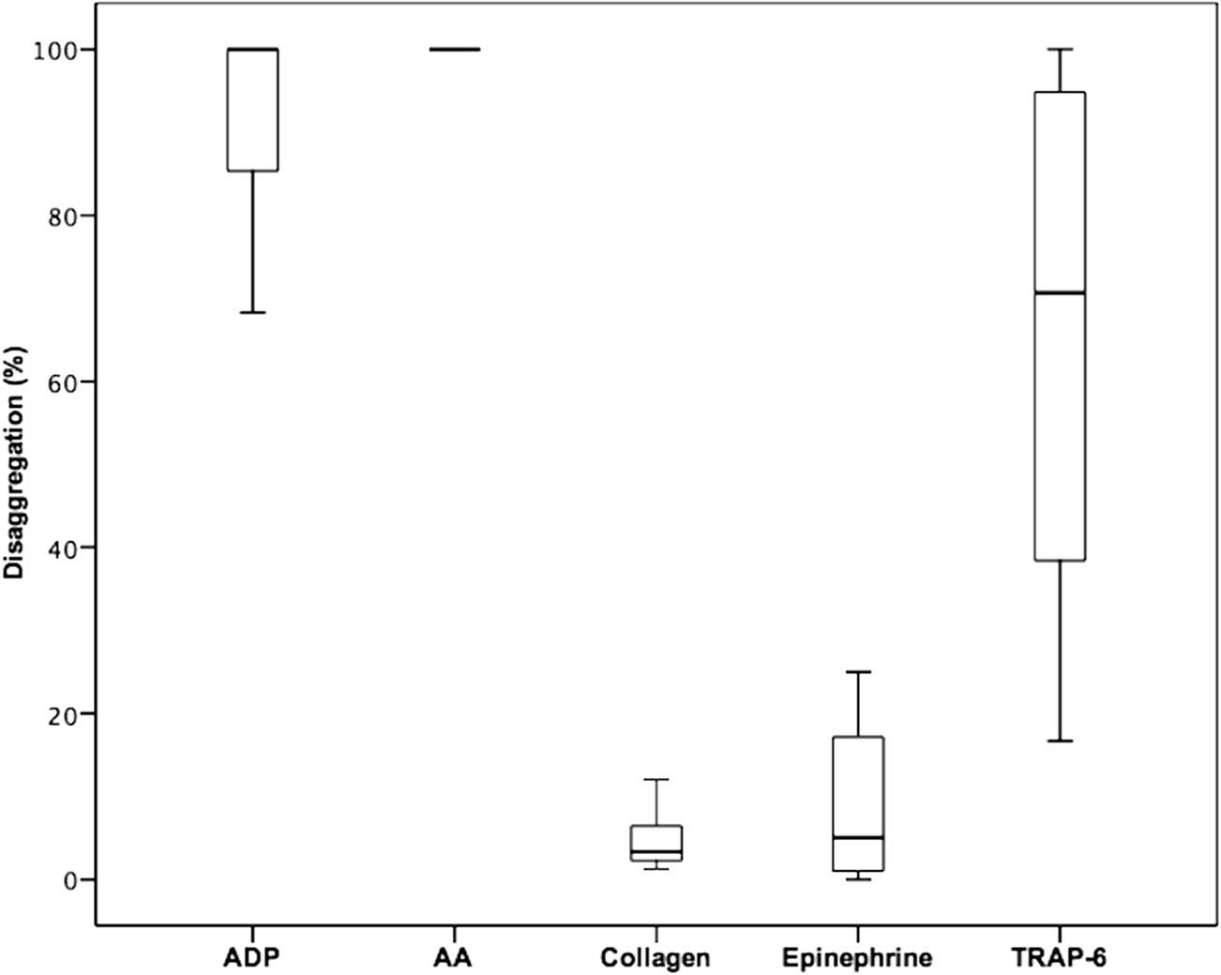
Disaggregation values obtained after stimulation with the agonists arachidonic acid (AA), adenosine diphosphate (ADP), collagen epinephrine, and thrombin receptor-activating peptide-6 (TRAP-6) in patients on aspirin and ticagrelor therapy (*n* = 20). The *boundaries of the box* show the lower and upper quartile of data, and the *line inside the box* represents the median. *Whisker*s are drawn from the edge of the box to the highest and lowest values that are outside the box but within 1.5 times the box length.

As seen in group 1, significant differences were noted between maximal and final aggregation values using all agonists (Tables 3 and 4). Median (interquartile range) values of disaggregation were highest for arachidonic acid (100% (100–100%) for prasugrel and ticagrelor) and ADP (100% (81.3–100%) for prasugrel and 100% (85.0–100%) for ticagrelor; Figs. 2 and 3). High disaggregation values were also noted using the agonist TRAP-6 (prasugrel 46.6% (23.5–77.6%); ticagrelor 70.7% (38.3–97.4%); Figs. 2 and 3). In contrast, for epinephrine, a difference of ≤5% (prasugrel 4.8% (0.0–12.5%); ticagrelor 5.0% (0.5–17.4%)) was seen. The lowest disaggregation rate was observed in response to collagen (prasugrel 3.0% (2.1–5.5%), ticagrelor 3.3% (2.2–6.5%); Figs. 2 and 3).

**Table 3:** Maximal and final platelet aggregation in response to adenosine diphosphate (ADP), arachidonic acid (AA), collagen, epinephrine, and thrombin receptor-activating peptide (TRAP)-6 in prasugrel-treated patients (*n* = 47)

	Maximal aggregation (%)	Final aggregation (%)	*p* value
ADP	26 (17–31)	0 (0–7)	<0.001
AA	2 (2–3)	0 (0–0)	<0.001
Collagen	57 (27–83)	55 (25–81)	<0.001
Epinephrine	19 (11–29)	16 (9–26)	<0.001
TRAP-6	70 (54–81)	38 (13–56)	<0.001

Data are presented as median and interquartile range

**Table 4:** Maximal and final platelet aggregation in response to adenosine diphosphate (ADP), arachidonic acid (AA), collagen, epinephrine, and thrombin receptor-activating peptide (TRAP)-6 in ticagrelor-treated patients (*n* = 20)

	Maximal aggregation (%)	Final aggregation (%)	*p* value
ADP	22.5 (16.3–28.5)	0 (0–7.5)	<0.001
AA	2 (1.3–4)	0 (0–0)	<0.001
Collagen	53 (28.8–78.8)	51.5 (27–76)	<0.001
Epinephrine	16 (8.5–27)	14.5 (8–26)	<0.001
TRAP-6	63.5 (46.3–77.5)	20 (1.5–43.8)	<0.001

Data are presented as median and interquartile range

In group 2, the extent of disaggregation varied significantly between all agonists (all *p* < 0.05), except between ADP and AA (*p* = 0.6). In cohort 3, significant differences were noted between all agonists (all *p* < 0.05) except between ADP and AA (*p* = 0.7) and between epinephrine and collagen (*p* = 0.1).

Disaggregation following the activation with ADP, AA, and TRAP-6 correlated inversely with the respective final aggregation values (prasugrel: ADP: *r* = −1 (*p* < 0.001); ARA: *r* = −0.8 (*p* < 0.001); TRAP-6: *r* = −0.9, (*p* < 0.001); ticagrelor: ADP: *r* = −1 (*p* < 0.001), AA: *r* = −0.6 (*p* = 0.01), TRAP-6: *r* = −0.9, (*p* < 0.001)).

In groups 2 and 3, HRPR occurred only in one person treated with prasugrel.

### Differences in Disaggregation Between Patient Groups

**Fig. 4:**
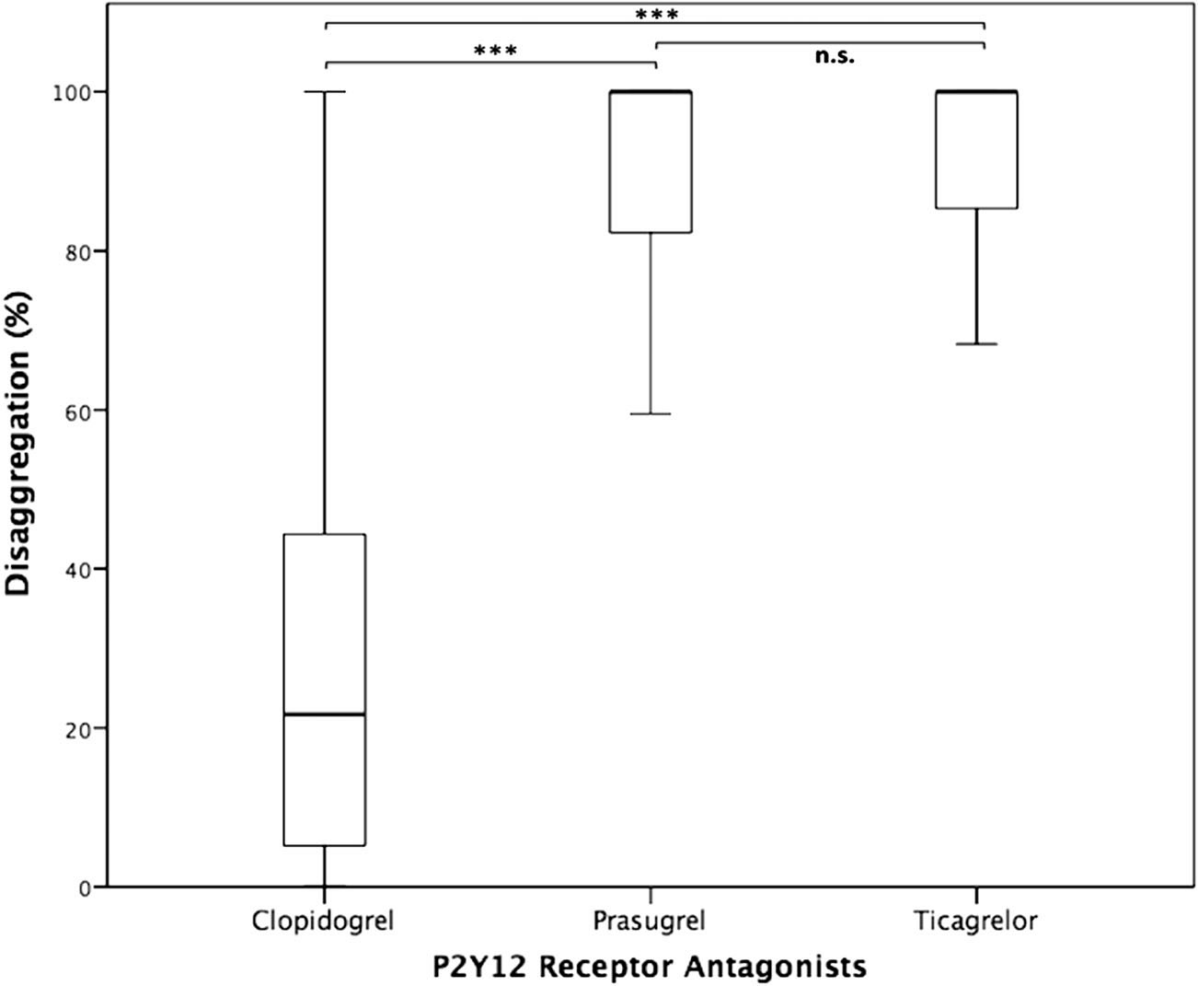
Disaggregation values using ADP as agonist in clopidogrel- (*n* = 256), prasugrel- (*n* = 47), and ticagrelor-treated (*n* = 20) patients The *boundaries of the box* show the lower and upper quartile of data, and the *line inside the box* represents the median. *Whiskers* are drawn from the edge of the box to the highest and lowest values that are outside the box but within 1.5 times the box length. ***p < 0.001

Disaggregation after platelet stimulation with ADP was significantly different between clopidogrel and prasugrel- or ticagrelor-treated patients (both *p* < 0.001), whereas it was similar between patients with ticagrelor and prasugrel therapy (Fig. 4, *p* > 0.05). Furthermore, disaggregation after platelet stimulation with AA, collagen, epinephrine, and TRAP-6 differed significantly between clopidogrel- and prasugrel-treated patients (all *p* < 0.001). Comparing clopidogrel and ticagrelor therapy, a significant difference in disaggregation could be obtained with use of all agonists (all *p* < 0.05). There were no significant differences of disaggregation between patients treated with prasugrel or ticagrelor.

## Discussion

Our study is the first to assess the stability of platelet aggregate formation in response to different agonists in patients on DAPT with aspirin and clopidogrel, prasugrel, or ticagrelor after angioplasty and stenting for cardiovascular disease. We found high levels of disaggregation after platelet stimulation with arachidonic acid or adenosine diphosphate in all three patient groups. In contrast, low disaggregation was observed after platelet stimulation with collagen and thrombin receptor-activating peptide-6 in clopidogrel-treated patients, and after platelet stimulation with collagen and epinephrine in prasugrel- and ticagrelor-treated patients.

Despite the development of new platelet function tests, LTA is still one of the most frequently used systems for the assessment of platelet function [6, 7] and residual platelet reactivity to ADP by LTA has repeatedly been linked to the occurrence of adverse ischemic events following PCI with stent implantation. [9, 11] We decided to measure on-treatment response to various agonists with this method because only by LTA maximal and final platelet aggregation as well as the level of disaggregation of platelet rich-plasma can be assessed. [4, 6, 12] We assessed disaggregation in all clopidogrel-treated patients 24 h and in all prasugrel- and ticagrelor-treated patients 72 h after the intervention. Thereby, all tests were performed at least 24 h after the administration of the clopidogrel loading dose and at least 72 h after the administration of the prasugrel or ticagrelor loading dose. Although these points of time may not be the final steady state, the major antiplatelet effect of clopidogrel [13, 14], prasugrel, and ticagrelor [15] should have occurred. The prolonged time interval between acute coronary interventions and blood sampling was chosen to avoid an influence of acute coronary syndromes (ACS) on the test results since previous studies have shown increased platelet activation in ACS. [16, 17]

The level of disaggregation may be of clinical relevance, as it may better mirror in vivo platelet activity. Consequently, the assessment of platelet disaggregation may allow a more precise risk stratification of patients with HRPR in those with high and low disaggregation, respectively. Patients with HRPR but high disaggregation may not be at an increased risk for adverse ischemic outcomes, whereas those with HRPR and low disaggregation may represent a particular high-risk population. Among the clopidogrel-treated patients in our study population, only 1 out of 39 patients with HRPR (2.6%) showed high disaggregation, whereas 27 patients with HRPR (69.2%) showed low disaggregation. However, it remains to be established if low disaggregation is associated with a further increase in cardiovascular risk and if therapeutic adjustments, e.g., switching from clopidogrel to prasugrel or ticagrelor, can ensure a better prognosis in these patients. Indeed, we observed almost complete disaggregation in all prasugrel- and ticagrelor-treated patients, which may contribute to their lower ischemic event rates but higher bleeding rates compared to patients receiving clopidogrel. [18, 19]

LTA is mostly performed using ADP with a final concentration of 20 μM as agonist. It should be noted, however, that 20 μM ADP is a rather high concentration that does not allow to distinguish between high and low platelet reactivity in healthy individuals anymore. [20] We therefore chose a concentration of agonists that allows to differentiate between low and high platelet reactivity in healthy individuals, and therefore would embrace also moderately inhibited platelets by their persistent aggregation.

In our study, disaggregation was highest using the agonists arachidonic acid and ADP. Aspirin as well as clopidogrel and prasugrel exert irreversible platelet inhibition, which may explain the high rates of disaggregation following stimulation with these agonists. [21, 22] Ticagrelor as a third ADP receptor antagonist acts directly and has previously been shown to more effectively block ADP-induced platelet aggregation compared to clopidogrel. [23] Apparently, its inhibitory activity is also associated with high levels of disaggregation. To the best of our knowledge, however, this is the first study comparing the pharmacodynamic effects of prasugrel and ticagrelor in humans. In rats, the inhibitory effect of these drugs on thrombus formation is similar. [24] Our data support these findings showing similar levels of aggregation and disaggregation of human platelets, which were inhibited by prasugrel or ticagrelor.

Previous studies have shown a great interindividual variability of clopidogrel-mediated platelet inhibition. In detail, loss-of-function polymorphisms of the cytochrome P450 isoenzymes CYP2C9 and CYP2C19, advanced age, diabetes, and concomitant treatment with morphine, calcium channel blockers, or the proton pump inhibitor omeprazole were associated with an attenuated response to clopidogrel therapy. [25] The latter was linked to an increased risk of adverse ischemic outcomes following PCI. [26] On the other hand, some studies suggested that smoking may enhance clopidogrel-mediated platelet inhibition. [27, 28] As previously published, we found higher levels of adenosine diphosphate inducible platelet aggregation by light transmission aggregometry in clopidogrel-treated patients carrying loss-of-function polymorphisms of CYP2C19 [29], in those aged 75 years or older [30], in those concomitantly receiving calcium channel blockers [31], and in those with chronic kidney disease [32] (all *p* < 0.05).

Another important clinical aspect is that low disaggregation in clopidogrel-treated patients may be an independent predictor of minor benefit from dose elevation. [33] In detail, Aradi D. et al. stratified patients undergoing emergency or elective PCI after a 600-mg loading dose of clopidogrel by maximal aggregation levels greater (defined as HRPR) or below 34% (no HRPR). Subsequently, patients with HRPR were treated with 150 mg clopidogrel/day, whereas patients without HRPR received 75 mg clopidogrel/day for 30 days. The elevated maintenance dose of 150 mg enhanced antiplatelet potency, but suboptimal clopidogrel response was still present in the majority of patients with baseline HRPR and could be predicted by initial platelet disaggregation below 16.5%. [33] However, this may not be relevant in prasugrel- and ticagrelor-treated patients since we observed 100% disaggregation in patients receiving the newer ADP receptor inhibitors.

In our study, we observed significant differences between maximal and final aggregation using all four agonists. Disaggregation was lowest with use of epinephrine, collagen, and TRAP-6. These results give new insights into platelet physiology, especially on possible alternative pathways of residual platelet activation during dual antiplatelet therapy. Further, our findings are consistent with prior analyses of our study group showing high activatability of platelets through PAR-1 and PAR-4 in patients on conventional DAPT. [34, 35] Moreover, we previously identified PAR-1-mediated platelet activation as a risk factor for adverse events after peripheral angioplasty with stent implantation. [36].

Besides the PAR pathways and in line with prior publications [37], our findings raise the question if there is synergistic platelet activation by collagen and thrombin involved in the development of re-thrombosis. Platelet activation by collagen can be induced through altering of blood flow patterns and shear forces. [38] There are different receptors for collagen including integrin α2β1 and glycoprotein VI and recent publications suggest cross-talk between PAR- and collagen-induced pathways. [37, 39, 40] Further studies with a long-term follow-up will lead to a better understanding of the association between stability of platelet aggregation and clinical outcome.

A limitation of our study is the lack of clinical outcome data. Moreover, we observed differences in baseline characteristics between clopidogrel-, prasugrel-, and ticagrelor-treated patients, which may have influenced the results of our study.

In conclusion, the stability of platelet aggregation in clopidogrel-treated patients was highest following collagen- and TRAP-6-stimulated platelet activation, whereas prasugrel and ticagrelor therapy were associated with low disaggregation after platelet activation with collagen and epinephrine. Our findings indicate that pathways of platelet activation that are not inhibited by aspirin and an ADP P2Y12 receptor antagonist allow persistent platelet aggregation in patients on DAPT and may therefore at least in part be responsible for adverse ischemic events during antithrombotic secondary prevention.

## Abbreviations

AA: Arachidonic acid
ADP: Adenosine diphosphate
DAPT: Dual antiplatelet therapy
HRPR: High on-treatment residual platelet reactivity
LTA: Light transmission aggregometry
PCI: Percutaneous coronary intervention
PPP: Platelet-poor plasma
PRP: Platelet-rich plasma
TRAP: Thrombin receptor-activating peptide
